# *Trypanosoma cruzi* CYP51 Inhibitor Derived from a *Mycobacterium tuberculosis* Screen Hit

**DOI:** 10.1371/journal.pntd.0000372

**Published:** 2009-02-03

**Authors:** Chiung-Kuang Chen, Patricia S. Doyle, Liudmila V. Yermalitskaya, Zachary B. Mackey, Kenny K. H. Ang, James H. McKerrow, Larissa M. Podust

**Affiliations:** 1 Department of Pharmaceutical Chemistry, University of California, San Francisco, California, United States of America; 2 Sandler Center for Basic Research in Parasitic Diseases, University of California, San Francisco, California, United States of America; 3 Department of Pharmacology, Vanderbilt University, Nashville, Tennessee, United States of America; McGill University, Canada

## Abstract

**Background:**

The two front-line drugs for chronic *Trypanosoma cruzi* infections are limited by adverse side-effects and declining efficacy. One potential new target for Chagas' disease chemotherapy is sterol 14α-demethylase (CYP51), a cytochrome P450 enzyme involved in biosynthesis of membrane sterols.

**Methodology/Principal Finding:**

In a screening effort targeting *Mycobacterium tuberculosis* CYP51 (CYP51_Mt_), we previously identified the *N*-[4-pyridyl]-formamide moiety as a building block capable of delivering a variety of chemotypes into the CYP51 active site. In that work, the binding modes of several second generation compounds carrying this scaffold were determined by high-resolution co-crystal structures with CYP51_Mt_. Subsequent assays against the CYP51 orthologue in *T. cruzi*, CYP51_Tc_, demonstrated that two of the compounds tested in the earlier effort bound tightly to this enzyme. Both were tested *in vitro* for inhibitory effects against *T. cruzi* and the related protozoan parasite *Trypanosoma brucei*, the causative agent of African sleeping sickness. One of the compounds had potent, selective anti–*T. cruzi* activity in infected mouse macrophages. Cure of treated host cells was confirmed by prolonged incubation in the absence of the inhibiting compound. Discrimination between *T. cruzi* and *T. brucei* CYP51 by the inhibitor was largely based on the variability (phenylalanine versus isoleucine) of a single residue at a critical position in the active site.

**Conclusions/Significance:**

CYP51_Mt_-based crystal structure analysis revealed that the functional groups of the two tightly bound compounds are likely to occupy different spaces in the CYP51 active site, suggesting the possibility of combining the beneficial features of both inhibitors in a third generation of compounds to achieve more potent and selective inhibition of CYP51_Tc_.

## Author Summary

Enzyme sterol 14α-demethylase (CYP51) is a well-established target for anti-fungal therapy and is a prospective target for Chagas' disease therapy. We previously identified a chemical scaffold capable of delivering a variety of chemical structures into the CYP51 active site. In this work the binding modes of several second generation compounds carrying this scaffold were determined in high-resolution co-crystal structures with CYP51 of *Mycobacterium tuberculosis*. Subsequent assays against CYP51 in *Trypanosoma cruzi*, the agent of Chagas' disease, demonstrated that two of the compounds bound tightly to the enzyme. Both were tested for inhibitory effects against *T. cruzi* and the related protozoan parasite *Trypanosoma brucei*. One of the compounds had potent, selective anti–*T. cruzi* activity in infected mouse macrophages. This compound is currently being evaluated in animal models of Chagas' disease. Discrimination between *T. cruzi* and *T. brucei* CYP51 by the inhibitor was largely based on the variability of a single amino acid residue at a critical position in the active site. Our work is aimed at rational design of potent and highly selective CYP51 inhibitors with potential to become therapeutic drugs. Drug selectivity to prevent host–pathogen cross-reactivity is pharmacologically important, because CYP51 is present in human host.

## Introduction

The drug development pipeline targeting diseases caused by trypanosome parasites is sparse [1]. Despite significant advances in its control over the last 15 years [2], Chagas' disease, caused by the parasitic protozoan *Trypanosoma cruzi* [3], remains a major public health concern in Latin America, with an estimated total of 8 million people infected [4]. Nifurtimox and benznidazole, the two principal drugs for treatment of Chagas' disease, were launched in 1967 and 1972 respectively, and suffer from the twin liabilities of serious side-effects and reduced efficacy in chronic *T. cruzi* infections [2]. A potential new target for Chagas' disease chemotherapy is sterol 14α-demethylase (CYP51) [5], a cytochrome P450 heme thiolate-containing enzyme which is involved in biosynthesis of membrane sterols in all biological kingdoms from bacteria to animals [6]. *T. cruzi* sterols are similar in composition to those in fungi, with ergosterol and ergosterol-like sterols the major membrane components [7]. Clinically employed antifungal azoles [8],[9] inhibit ergosterol biosynthesis in fungi and are partially effective against *Leishmania* and *Trypanosoma* parasites [10]–[12]. Azoles block CYP51 activity, resulting in decline of the normal complement of endogenous sterols and accumulation of various 14α-methyl sterols with cytostatic or cytoxic consequences [11]. Aside from the compounds optimized for antifungal therapy, other CYP51 inhibitors with strong anti-*T. cruzi* activity have also been reported [13]–[15].

Mammalian CYP51 shares relatively modest overall sequence identity – below 30% – with its fungal and protozoan counterparts, but within the active site the amino acid residues are far more conserved. Based upon crystal structures of CYP51 of *M. tuberculosis* (CYP51_Mt_) [16]–[20], three of the thirteen active site residues, Y76, F83, and H259 (numbering according to CYP51_Mt_), are invariant throughout the *cyp51* gene family. Two residues, F78 and F255, are specific to the methylation status of the C-4 atom in the sterol nucleus [18],[21], and amino acid identities of seven other positions strongly overlap across phyla [19],[20]. Of the thirteen residues, only one, R96, seems to be phylum-specific. This similarity confines design of selective CYP51 inhibitors to a species-specific cavity in the active site defined by the hydrophobic residues F78, L321, I322, I323, M433, and V434.

To discover novel inhibitors, we previously screened a library of small synthetic molecules against the CYP51_Mt_ target [19]. The *N*-[4-pyridyl]-formamide moiety of the top hit, α-ethyl-N-4-pyridinyl-benzeneacetamide (EPBA), was found to bind unvaryingly in the CYP51 active site with Y76, H259, and the heme prosthetic group. The uniformity of interactions with CYP51 suggested that this scaffold could be used to target a variety of chemotypes to the active site. To verify this assumption, we determined the binding modes of second generation compounds containing the *N*-[4-pyridyl]-formamide moiety by determining their co-crystal structures with CYP51_Mt_. We also spectrally characterized binding of these compounds to CYP51 of both *T. cruzi* (CYP51_Tc_) and the related protozoan parasite *T. brucei*, the causative agent of African sleeping sickness, (CYP51_Tb_). Two compounds were selected based on their nanomolar binding affinities toward CYP51_Tc_ and subsequently tested *in vitro* for inhibitory effects against both pathogens. One of the two compounds revealed potent and selective inhibitory effect against *T. cruzi* infection in mouse macrophage cells.

## Methods

### Preparation of CYP51_Mt_

CYP51_Mt_ double C37L/C442A and triple C37L/F78L/C442A mutants were prepared as described elsewhere [19]. The surface exposed cystein residues C37 and C442 were removed via replacement with leucine and alanine, respectively, to improve protein homogeneity and aid crystallization [18]. The functionally important F78 in the active site was replaced in the triple mutant by leucine, which invariantly occupies this position in the mammalian CYP51 isoforms.

### Preparation of CYP51_Tc_

Design of the CYP51_Tc_ expression vector was based on an entity in the NCBI data bank (ID AY283022 [22]), which was modified by replacing the first 31 residues upstream of Pro32 with the fragment MA**KKTSSKGKL** from the CYP2C3 sequence [23] (CYP2C3 residues marked in bold) to improve protein solubility, and by inserting a His_6_-tag at the C-terminus to facilitate purification. This coding sequence (kindly provided by M. Waterman in the form of the pET vector) was subsequently sub-cloned into pCWori vector [24] between the NdeI and HindIII restriction sites and in this form used to transform *Escherichia coli* strain HMS174(DE3).

**Figure 1:**
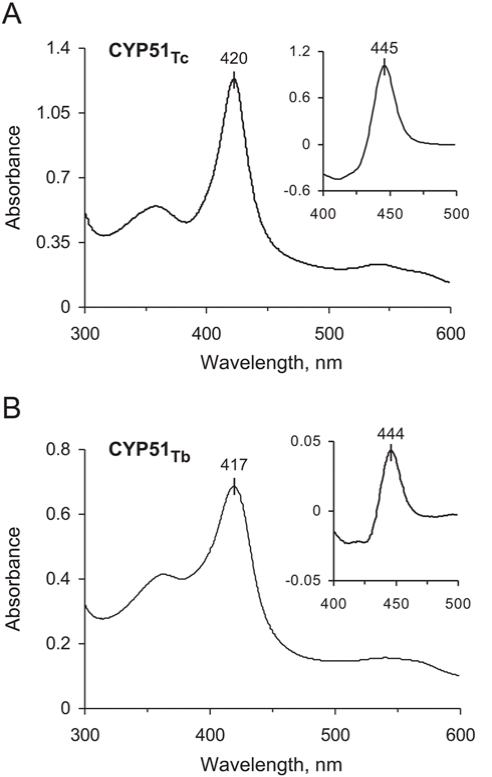
Spectral characteristics of CYP51_Tc_ (A) and CYP51_Tb_ (B). Main panel shows the absolute protein spectrum, while insert shows CO-bound reduced difference spectrum.

Transformants were grown for 5 h at 37°C and 250 rpm agitation in Terrific Broth medium supplemented with 1 mM thiamine, 50 µg/ml ampicillin, and trace elements. CYP51_Tc_ expression was induced by the addition of isopropyl-β-D-thiogalactopyranoside (IPTG, final concentration 0.2 mM) and δ-aminolevulinic acid, a precursor of heme biosynthesis (final concentration 1 mM). Following induction, temperature was decreased to 25°C and agitation to 180 rpm. After 30 hours the cells were harvested and lysed by sonication. Insoluble material was removed from crude extract by centrifugation (30 min at 35,000 rpm). The supernatant was subjected to a series of chromatographic steps, including nickel-nitrilotriacetic acid (Ni-NTA) agarose (QIAGEN), followed by Q-Sepharose (Amersham Biosciences) in the flow-through regime, and then by S-Sepharose (Amersham Biosciences). From the S-Sepharose, protein was eluted in a 0.2 to 1.0 M NaCl gradient and observed by means of a 12% SDS-PAGE to be virtually homogeneous. Fractions containing P450 were combined, concentrated using a Centriprep concentrating device (Millipore), and stored at −80°C. Twenty mM Tris-HCl, pH 7.5, 10% glycerol, 0.5 mM EDTA, and 1 mM DTT were maintained throughout all chromatographic steps. Spectral characteristics of CYP51_Tc_ are shown in Figure 1A.

### Preparation of CYP51_Tb_

The expression vector for CYP51_Tb_ (ID EAN79583) was generated using *T. brucei* genomic DNA and upsteam GCGCGCATATG **GCT**CTTGAAGTTGCC and downstream CGCAAGCTTCTA **GTGATGGTGATGGTGATGGTGATG**AGCAGCTGCCGCCTTCC primers. The underlining denotes an NdeI restriction cloning site in the upstream primer and the HindIII restriction cloning site in the downstream primer followed by the stop codon. The bold sequence in the upstream primer highlights second codon replaced with alanine to optimize expression in *E. coli* cells [24]. The boldface in the downstream primer indicates the His_8_ tag. The original genomic DNA contained internal NdeI site at 345 base pair which was removed by introducing a silent mutation via the quick-change mutagenesis protocol (Stratagene). DNA amplification reaction was carried out as follows: 5 min at 94°C, annealing for 1 min at 55°C, and extension for 1 min at 72°C, for 30 cycles, followed by extension for 10 min at 72°C. The purified 1.5 kb PCR product was ligated into the pCR 2.1 TA cloning vector (Invitrogen). Insert was subsequently cleaved with NdeI and HindIII and ligated into pCWori vector digested with the same restriction enzymes and treated with alkaline phosphatase. The identity of the resulting vector was confirmed by DNA sequencing.

*E. coli* HMS174(DE3) strain was co-transformed with this vector and the pGro7 plasmid (Takara) encoding the *E. coli* chaperones GroES and GroEL. Double transformants were selected on agar plates containing both ampicilin and chloramphenicol. One liter of Terrific Broth medium supplemented with 1 mM thiamine, 100 µg/ml ampicillin, 40 µg/ml chloramphenicol, and trace elements was inoculated with 10 ml of overnight culture and growth continued at 37°C and 250 rpm agitation until OD_600_ reached 0.3. At that point expression of chaperones was induced with 0.2% arabinose. Growth continued at 27°C and 180 rpm until OD reached 0.6. Then CYP51_Tb_ expression was induced by the addition of isopropyl-β-D-thiogalactopyranoside (IPTG, final concentration 0.3 mM), and δ-aminolevulinic acid (1 mM). Following induction, temperature was decreased to 15°C. After 48 hours the cells were harvested and lysed by sonication. Purification was conducted similarly to as described above for CYP51_Tc_, with the qualification that S-Sepharose was used in the flow-through regime, while the protein was bound to and eluted from the Q-Sepharose column. Spectral characteristics of CYP51_Tb_ are shown in Figure 1B.

### Crystallization, Data Collection, and Determination of Crystal Structure

**Figure 2:**
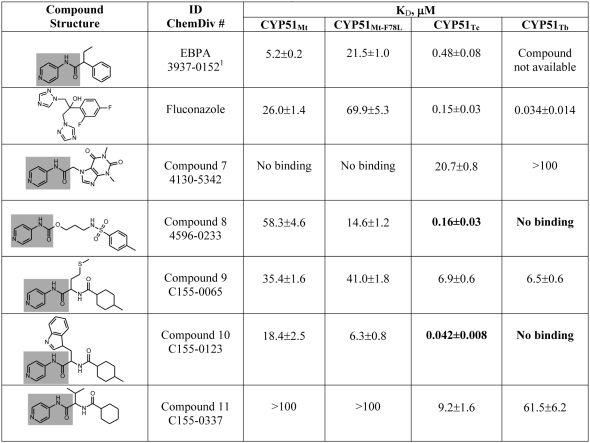
Chemical structures and binding affinities of compounds. ^1^Compounds are identified by the numbers in the ChemDiv, Inc., product library catalog.

Five compounds (Fig. 2), purchased from ChemDiv (San Diego, California) were used for co-crystallization with the CYP51_Mt_ C37L/C442A double mutant. Compared to the wild type, this construct has superior propensity for crystallization. Compound numbering is according to the order in which they were received in our laboratory, with number **7** being the first used in the current work. Ligands were dissolved in Me_2_SO at ≤100 mM stock concentration, and brought to final concentrations ranging from 1 to 5 mM in the crystallization mix, depending on ligand solubility. Protein concentration was 0.2 mM. A narrow crystallization screening grid (15–30% PEG 4000, 2–12% isopropanol, 0.1 M HEPES, pH 7.5), previously devised to obtain CYP51_Mt_ crystals [16],[18],[19] was utilized for co-crystallization of complexes by the vapor diffusion hanging drop method. Four co-crystal forms were obtained, all diffracted to resolutions between 1.56 to 1.60 Å. Diffraction data were collected at 100–110 K at the Southeast Regional Collaborative Access Team (SER-CAT) 22ID beamline, Advanced Photon Source, Argonne National Laboratory using SER-CAT mail-in data collection program (Table 1). The images were integrated and the intensities merged with the HKL2000 software suite [25]. The structures were determined by molecular replacement using coordinates of estriol-bound CYP51_Mt_ (Protein Data Bank ID 1X8V) as a search model. The final atomic models were obtained after a few iterations of refinement using REFMAC5 [26] and model-building using the COOT graphics modeling program [27]. The quality of the structures was assessed by the program PROCHECK [28]. One residue, A46, was found in the generously allowed region of the Ramachandran plot in all structures where, together with the adjacent G47, it enables a sharp turn between two β strands.

**Table 1:** Crystallographic data and statistics.

Compound #	Compound 8	Compound 9	Compound 11
ChemDiv #	4596-0233	C155-0065	C155-0337
PDB ID	2W0B	2W09	2W0A
**Data collection**			
Wavelength, Å	1.0000	1.0000	1.0000
Resolution, Å	1.56	1.57	1.60
Unique reflections	63021	59811	53704
Redundancy^a^	5.5 (4.7)	5.4 (3.4)	5.1 (3.9)
Completness, %	99.5 (99.1)	95.8 (79.4)	93.8 (77.7)
Space group	P2_1_2_1_2_1_	P2_1_2_1_2_1_	P2_1_2_1_2_1_
Cell dimensions (a, b, c), Å	46.7, 84.8, 110.3	46.4, 85.1, 110.9	44.7, 85.7, 110.9
Molecules in asymmetric unit	1	1	1
Solvent content, %	40	40	40
R_sym_ b, %	5.2 (29.7)	7.7 (32.8)	7.0 (32.2)
I/σ	32.1 (4.3)	45.5 (4.4)	32.5 (3.4)
**Refinement**			
Reflections used in refinement	62962	59743	53638
R_cryst_ (R_free_)^c^, %	16.4/20.1	18.8/22.2	18.9/23.2
No. of atoms	3989	3753	3726
Protein	3542	3409	3376
Heme	43	43	43
Substrate	24	24	22
Water	380	277	285
Wilson plot B-values, Å^2^	15.2	22.6	18.7
Mean B-factor, Å^2^	14.6	25.5	20.3
Protein	14.0	24.9	19.7
Heme	9.6	22.6	21.0
Substrate	14.9	29.0	21.0
Water	22.8	32.6	27.8
r.m.s. deviations			
Bond length, Å	0.010	0.013	0.013
Bond angles, °	1.3	1.4	1.4
Ramachandran (%)^d^	91.9/7.9/0.3	91.7/8.0/0.3	91.5/8.2/0.3

a Numbers in parentheses correspond to the highest resolution shell.

b R_sym_ = Σ|*I_i_*−〈*I*〉|/Σ*I_i_*, where *I_i_* is the intensity of the *i* ^th^ observation, and 〈*I*〉 is the mean intensity of reflection.

c R_cryst_ = Σ∥Fo|−|Fc∥/Σ|Fo|, calculated with the working reflection set. R_free_ is the same as R_cryst_ but calculated with the reserved reflection set.

d Program PROCHECK [28], portions of the protein residues in most favored/additional allowed/generously allowed regions.

### Spectroscopic Binding Assays

Spectroscopic binding assays were performed at room temperature in 1-ml quartz cuvette containing 1 µM or 2 µM CYP51 in 50 mM Tris-HCl, pH 7.5, and 10% glycerol using a Cary UV-visible scanning spectrophotometer (Varian). Concentration of CYP51 was determined at 450 nm from the difference spectra between the carbon monoxide-bound ferrous and water-bound ferric forms, with an extinction coefficient of 91,000 M^−1^ cm^−1^ [29]. In the first round, compounds dissolved in Me_2_SO at 10 mM concentration were added to the 2 µM protein solution in 0.5 µl aliquots, resulting in concentration increases from 5 µM to 50 µM in 5 µM increments. The same amounts of Me_2_SO alone were added to the protein in the reference cuvette, followed by recording the difference spectra. In the second round, compounds with high affinities were diluted to 100 µM by Me_2_SO and titrated into 1 µM protein solution in 1 µl aliquots to increase compound concentration from 0.1 µM to 2 µM in 0.1 µM increments. To determine the K*_D_*, we used the GraphPad PRISM software (Graphpad Software Inc.) to fit titration data to either rectangular or quadratic hyperbolas to correct for the bound ligand fraction, according to the functions ΔA = (A_max_(S/K*_D_*+S) or ΔA = (A_max_/2[E])((K*_D_*+[E]+[L])−((K*_D_*+[E]+[L])^2^−4[E][L])^1/2^), respectively, where E is total enzyme and L total ligand concentration, A_max_ the maximal absorption shift at saturation, and K*_D_* the apparent dissociation constant for the enzyme-ligand complex.

### *T. cruzi* Assay

Irradiated (1000 rads) J774 mouse macrophages were plated in 12-well tissue culture plates 24 h prior to infection with 10^5^ *T. cruzi* Y strain trypomastigotes for 2 h at 37°C. Cultures were maintained in RPMI-1640 medium with 5% heat-inactivated fetal calf serum and 5% CO_2_ with the addition of 10 µM compound **8** or **10**. Untreated controls, controls treated with the inhibitor K11777 (10 µM) [30],[31], and uninfected macrophage controls were also included. All cultures were in triplicate and medium was replaced every 48 h. Treatment with CYP51 inhibitors continued for up to 27 days. Subsequently, treated cultures were maintained without inhibitor for an additional 13–15 days to confirm inhibitor effectiveness and cure of infected cells. Cultures were monitored daily by contrast phase microscopy to determine presence of *T. cruzi* infected cells and free infectious trypomastigotes (Table 2).

**Table 2:** *T. cruzi* infection *in vitro*.

Treatment	Host cell survival	*T. cruzi* development
Untreated control	5 days	5 days
Compound **8** (10 µM)	5 days	5 days
Compound **10** (10 µM)	40 days	No
K11777 control	40 days	No

### IC50

To determine IC50, mouse J774 macrophages were irradiated (1000 rads) to deter growth and plated onto 12-well tissue culture plates. Cells were infected with 10^5^ tissue culture trypomastigotes of the Y strain of *T. cruzi* for 2 h at 37°C, as described above. Next, medium was replaced with the addition of compound **10** at 0, 1 nM, 10 nM, 100 nM, 500 nM, 1 µM, 5 µM, and 10 µM; these cultures were incubated for 52 h at 37°C. Controls with 10 µM K11777 and 10 µM compound **8** were also included. All treatments were performed in triplicate to ensure statistical validity. Cultures were then fixed in 4% paraformaldehyde in PBS for 2 h at room temperature and stained with DAPI (10 nM) in PBS. One hundred cells and their intracellular parasites were quantified as previously described to estimate the mean number of parasites/cell [32]. Mean P/cell data were plotted against compound concentration to estimate the IC50.

### Mammalian Cell Toxicity Assay

Toxicity was evaluated in bovine muscle cells (BESM), mouse J774 macrophages, and human Huh7 hepatocytes against compound 10 at 10 µM, 50 µM and 100 µM concentrations. After 48 h in culture at 37°C, cells were stained with 10% Tripan Blue and the number of live *versus* dead cells was quantified (Table 3).

**Table 3:** Toxicity in mammalian cells.

Treatment	Mean Live Cells±SD		
	BESM (bovine)	Macrophages (mouse)	Hepatocytes (human)
Untreated control	89±5	97.5±0.8	92.5±0.8
Compound 10 (10 µM)	88.5±0.5	96±0.1	94±1
Compound 10 (50 mM)	62.5±0.8	91±0.1	83±3
Compound 10 (100 µM)	10±1	15±0.5	53±1

### *T. brucei* Assay

Trypanosomes were grown in complete HMI-9 medium containing 10% FBS, 10% Serum Plus medium (Sigma Inc. St. Louis Mo. USA) and 1× penicillin/streptomycin. Trypanosomes were diluted to 1.0×10^5^/ml in complete HMI-9 medium. Diluted trypanosomes were aliquoted in Greiner sterile 96-well flat white opaque culture plates using a WellMate cell dispenser (Matrix Tech., Hudson, NH, USA). Compounds **8** and **10** were serially diluted in Me_2_SO. Trypanosomes were incubated with the compounds for 48 h at 37°C with 5% CO_2_ before monitoring viability. Trypanosomes were then lysed in the wells by adding 50 µl of CellTiter-GloTM (Promega Inc., Madison, WI, USA). Lysed trypanosomes were placed on an orbital shaker at room temperature for 2 min. The resulting ATP-bioluminescence of the trypanosomes in the 96-well plates was measured at room temperature using an Analyst HT plate reader (Molecular Devices, Sunnyvale, CA, USA).

## Results

### Crystal Structures of CYP51_Mt_-Inhibitor Complexes

**Figure 3:**
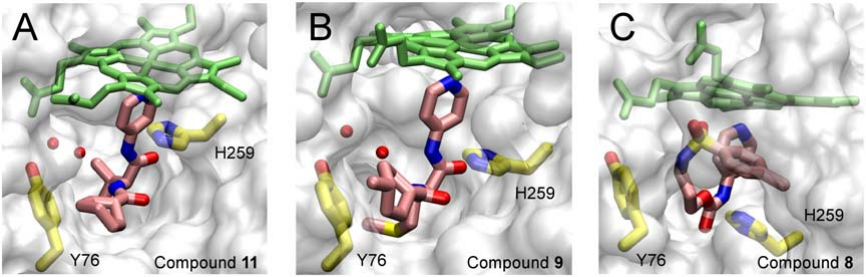
Overall view of compound binding in the active site. Compounds 11 (A), 9 (B), and 8 (C) (highlighted in *pink*) bound in the active site of CYP51_Mt_ are shown looking in from the active site opening. For clarity only one conformation of each compound is shown. Protein is represented by the semitransparent accessible surface (*gray*). The ordered BC-loop obstructs the view in the CYP51_Mt_-8 complex in (C). The invariable elements of the CYP51 active site, Y76, H259 (*yellow*), and heme (*green*), are in a stick mode. Water molecules are shown as *red spheres*. Oxygen atoms are *red*, nitrogen *blue*, sulfur *yellow*. Images were generated using the VMD program [35].

**Figure 4:**
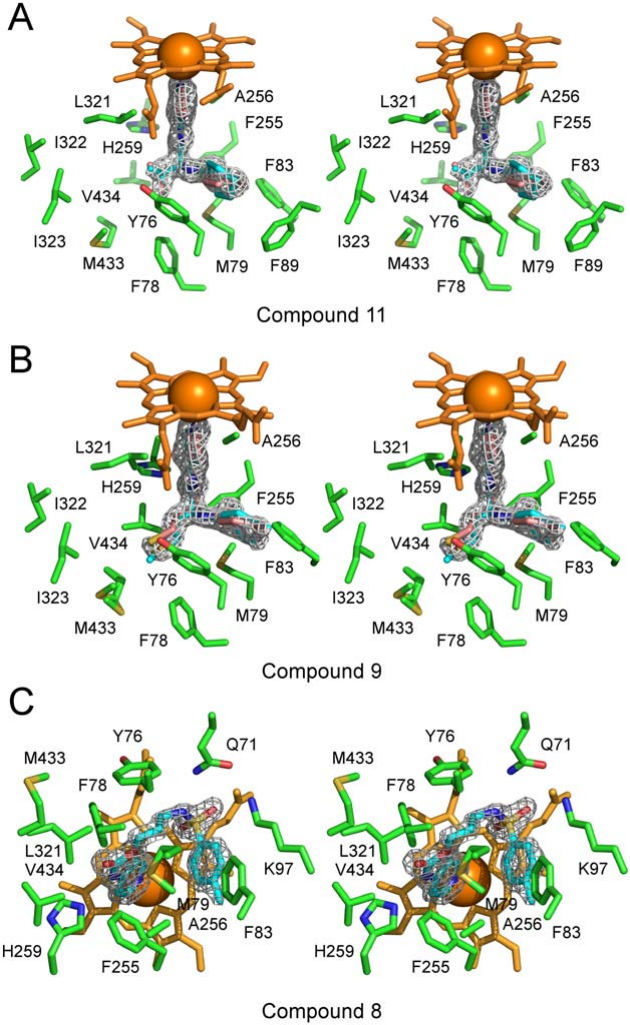
Stereo view of compounds in the active site. Compounds 11 (A), 9 (B), and 8 (C) are shown surrounded by the CYP51 active site residues. The fragments of the electron density 2Fo-Fc map (*gray mesh*) are cut at 1.2 σ. Different conformers in (A) and (B) are highlighted in *pink* and *cyan*. Images were generated using PYMOL [36].

Co-crystals were obtained for compounds **8**, **9** and **11**. Compound **10** failed to generate any crystals with CYP51_Mt_. Compound **7** was not found in the CYP51_Mt_ active site in the crystal, which is consistent with lack of spectrally detectable binding (Fig. 2). Compounds **8** (3-{[(4-methylphenyl)sulfonyl]amino}propylpyridin-4-ylcarbamate), **9** (cis-4-methyl-N-[(1S)-3-(methylsulfanyl)-1-(pyridin-4-ylcarbamoyl)propyl]cyclohexanecarboxamide), and **11** (N-[(1S)-2-methyl-1-(pyridin-4-ylcarbamoyl)propyl]cyclohexanecarboxamide), were observed bound in the CYP51_Mt_ active site as predicted, through the coordination of the heme iron via a lone pair of aromatic nitrogen electrons of the *N*-[4-pyridyl]-formamide moiety (highlighted in *gray* in Fig. 2) and interactions with the invariant residues Y76 and H259 (Fig. 3). Functional groups other than the *N*-[4-pyridyl]-formamide moiety in compounds **9** and **11** either were accommodated in the species-specific cavity or else protruded through the opening of the active site toward bulk solvent. H259 hydrogen-bonded to the carbonyl oxygen in both compounds, while interactions with Y76 were mediated by two similarly positioned water molecules (Figs. 3A and 3B). The residual F_o_-F_c_ electron density map suggested two alternative conformations for compounds **11** and **9**, designated by *pink* and *cyan* respectively in Figures 4A and 4B. In the CYP51_Mt_-compound **11** complex, the cyclohexane ring protruded toward the bulk solvent (Fig. 3A), barely interacting with the protein in two alternative conformations (Fig. 4A). Together with the limited interactions of the isopropyl moiety, this lack of contact explains the low binding affinity of **11**. In the CYP51_Mt_-compound **9** complex, the methylcyclohexane moiety protruded toward bulk solvent, while the methylsulfanyl group loosely bound in the species-specific cavity (Fig. 3B) in two alternative conformations (Fig. 4B). The side chain of M433 also adopted two alternative conformations. In both complexes, a portion of the BC-region was disordered and missing from the electron density map. Although racemic mixtures were used for co-crystallization, only one enantiomer of each compound was found in the active site.

A different binding mode was revealed for compound **8**. Its flexible backbone allowed it to fold head-to-tail over the heme plane to bring the methylphenylsulfonamide group into intramolecular stacking interactions with the pyridinyl moiety and also with the heme macrocycle (Fig. 3C, 4C). Folding minimized the nonpolar surface of compound **8** by exposing the sulfonamide group to interactions with Q72, K97, and the heme propionate side chain. The hydrophobic side chain of K97 aligned along the methylphenyl moiety. A similar folding of the benzothiadiazolsulfonamide group has been observed in previous work for 2-[(2,1,3-benzothiadiazol-4-sulfonamide]-2-phenyl-N-pyridin-4-acetamide (BSPPA) [19]. Mutually stabilizing protein-ligand interactions involving the BC-loop residues including F78 result in increased binding affinity of the CYP51_Mt_-compound **8** complex and in unambiguous electron density both for compound **8** (Fig. 3C) and for the entire BC-region. In the CYP51_Mt_-compound **8** complex, H259 directly H-bound to the amide nitrogen of compound **8**, whereas Y76 interacted hydrophobically with the compound's flexible backbone (Fig. 3C).

### Binding Affinities of CYP51 Ligands

**Figure 5:**
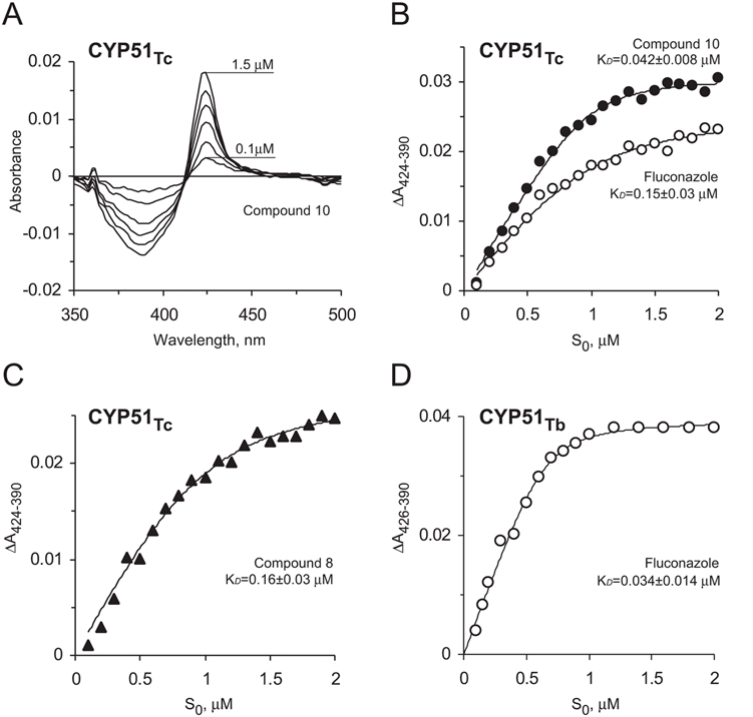
Spectroscopic binding of compounds. (A) Type II spectral responses of CYP51_Tc_ to increasing concentrations of compound 10. The concentration dependence of compound 10, fluconazole (B), and compound 8 (C) binding were deduced from the difference absorption changes obtained from the titration of CYP51_Tc_ with increasing concentrations of the inhibitor. The concentration dependence of fluconazole (D) was deduced from the difference absorption changes obtained from the titration of CYP51_Tb_.

Binding affinities of all five compounds were examined against both wild type and a ‘humanized’ F78L mutant form of CYP51_Mt_, CYP51_Tc_, and CYP51_Tb_ using spectroscopic assays (Fig. 5). These assays utilize the property of P450 enzymes to shift the ferric heme iron Soret band following replacement of a weak ligand, the water molecule, with a stronger one, the nitrogen-containing aromatic pyridinyl group (Fig. 5A). All compounds had markedly reduced or no binding affinity toward CYP51_Mt_, compared to the parental EPBA (Fig. 2). No binding was observed for compound **7**, while the K*_D_* for compound **11** exceeded 100 µM, indicating weak binding.

However, the binding affinity of all compounds examined, including compound **7**, was significantly higher to CYP51_Tc_ than to CYP51_Mt_. Remarkably, the binding affinities of compounds **8** and **10** to CYP51_Tc_ were 300- and at least 500-fold respectively higher, equaling or exceeding that of the antifungal CYP51 inhibitor fluconazole, which was used as a reference (Fig. 5B, C). A K*_D_* of at least 40 nM was estimated for compound **10** by spectral assays, with the binding curve reaching a plateau at about a 1∶1 protein to ligand ratio. This value strongly suggests that the K*_D_* must be notably higher, although further dilution of protein in an attempt to obtain a more accurate value significantly decreased the quality of the spectra and this effort was thus abandoned. The IC50 of ∼1 nM for *T. cruzi* intracellular growth inhibition, determined for compound **10** as described below, may better reflect true K*_D_* value. Compounds **8** and **10**, which had highest binding affinity to CYP51_Tc_, were spectrally silent toward CYP51_Tb_, indicating no binding in the active site (Fig. 2). As expected, CYP51_Tb_ had nanomolar affinity for fluconazole (Fig. 5D), but again, the plateau was reached at a 1∶1 protein to inhibitor ratio, so the binding constant could not be determined more accurately. Compound **9** bound both CYP51_Tc_ and CYP51_Tb_ with the same affinity.

The K*_D_* values for compounds **8** and **10** slightly decreased for the F78L CYP51_Mt_ mutant compared to the wild type, while the K*_D_* values for the other compounds increased (Fig. 2).

### Inhibitory Effects against *T. cruzi*

With submicromolar affinities toward CYP51_Tc_ of 160 nM and <40 nM respectively, compounds **8** and **10** were examined *in vitro* for inhibitory effects against both *T. cruzi* and *T. brucei*. In a mouse macrophage assay, *T. cruzi* completed its intracellular development in 5 days in untreated controls, resulting in death of host macrophages and abundant trypomastigotes in culture supernatant (Table 2). As anticipated, the control compound K11777 [30] cured *T. cruzi* infection. No parasites survived a treatment regime of 27 days with compound **10**. Cure of host cells was confirmed by incubation of the cultures for an additional 15 days in the absence of inhibitor. In contrast, and similarly to untreated controls, *T. cruzi* completed its development in 5 days in cultures treated with compound **8**.

**Figure 6:**
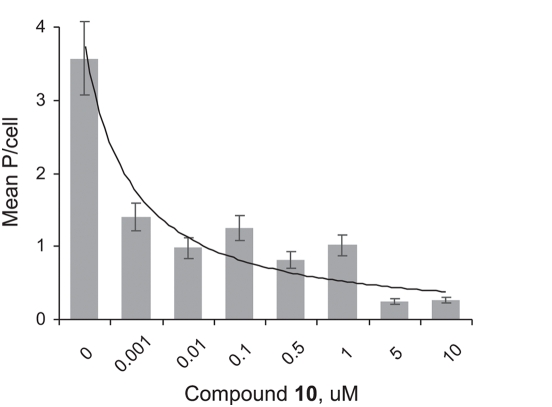
Inhibition of *T. cruzi* intracellular amastigotes by compound 10. *T. cruzi* intracellular multiplication was evaluated at 52 hr of incubation at several concentrations of the inhibitor by determining the number of parasites/cell. Intracellular parasites were counted per one hundred cells to estimate a mean number of parasites per cell. Approximation of concentration dependence of mean P/cell±SD data with a smooth curve highlights the 50% drop in parasite count at ∼1 nM compound 10. SD did not exceed 14% of the mean.

An IC50 of ∼1 nM concentration for compound **10** (Fig. 6) was estimated for *T. cruzi* intracellular amastigotes. *T. cruzi* developed well intracellularly in untreated macrophages with a final mean number of 3.57±0.5 P/cell (0% inhibition). As determined previously, 10 µM compound **10** was deleterious for *T. cruzi*, with a mean of 0.25±0.01 P/cell (100% growth inhibition). Ten µM of control compound K11777 was also parasiticidal for *T. cruzi* with a mean of 0.25±0.01 P/cell (IC100) [30], while compound **8** was not parasiticidal at this concentration with a mean of 1.22±0.1 P/cell (data not shown).

Toxicity for mammalian cells was addressed by treating the three different cell types with increasing concentrations of compound **10** (Table 3). No toxicity was observed at 10 µM compound **10**, while 50 µM was mildly toxic for muscle cells. One hundred µM compound **10** was toxic for all mammalian cells tested, especially muscle cells.

Consistent with the spectral binding assays, neither compound **8** nor **10** had any inhibitory effects against cultured *T. brucei* even at the highest tested concentration of 10 µM.

### Protein Data Bank Accession Numbers

The atomic coordinates and structure factors determined in this study (Protein Data Bank IDs 2W09, 2W0A, and 2W0B) have been deposited in the Protein Data Bank, Research Collaboratory for Structural Bioinformatics, Rutgers University, New Brunswick, NJ (http://www.rcsb.org/).

## Discussion

We explored sterol 14α-demethylase (CYP51) as a potential target for trypanosomiasis chemotherapy by probing CYP51_Mt_, CYP51_Tc_, and CYP51_Tc_ with second generation compounds that contain a universal building block, the *N*-[4-pyridyl]-formamide moiety, which is capable of delivering small molecule compounds to the CYP51 active site. The affinities of the *N*-[4-pyridyl]-formamide-derivative compounds that we tested against CYP51_Mt_ were lower than that of EPBA (Fig. 2), from which the formamide building block was derived. Affinities of all compounds examined were much higher toward CYP51_Tc_ than to CYP51_Mt_. Strikingly large increases in binding affinities – 300 and 500 fold – were observed for compounds **8** and **10**. Although compound **10** did not produce crystals with CYP51_Mt_, based on the binding modes of compounds **9** and **11**, we reason that the methylcyclohexanecarboxamide moiety of compound **10** protrudes toward the BC-loop, suggesting that the indole ring binds in the species-specific cavity, including the space occupied in CYP51_Mt_ by the F78 aromatic ring, which is absent from CYP51_Tc_ but present in CYP51_Tb_ and CYP51_Mt_. Consistent with this hypothesis, compound **10** selectively bound CYP51_Tc_, inhibited *T. cruzi* growth with the IC50 value close to the K*_D_* estimated in the spectral binding assays, and cured mouse macrophages infected with *T. cruzi* Y strain at 10 µM concentration without harming them.

In contrast, compound **10** failed to bind CYP51_Tb_ despite the identity of 12 of the 13 active site substrate binding residues, and 83% overall sequence identity between *T. cruzi* and *T. brucei* CYP51 orthologues. This result is a striking indication of the sensitivity of CYP51 to alterations of the topography of its active site at position 78. The difference in position 78 is of functional importance, because phenylalanine at this site is strictly specific to protozoa and plant species metabolizing 4α-methylated sterols [18]. Interestingly, *T. cruzi* is the only protozoan where the corresponding position (position 105 according to *T. cruzi* numbering) is occupied by isoleucine. Consistent with this observation, CYP51_Tc_ is catalytically more closely related to its fungal and animal orthologues, preferentially converting 4α,β-dimethylated sterol substrates [21], whereas *T. brucei* CYP51 is strictly specific to 4α-methylated obtusifoliol and norlanosterol [33]. The proteobacterium *Methylococcus capsulatus*, known to synthesize sterols from squalene [34], is the only other known organism having isoleucine in the CYP51 position corresponding to F78. Not surprising, compound **10** was inactive against *T. brucei* in inhibitory assays *in vitro*.

In humans and animals metabolizing 4α,β-dimethylated 24,25-dihydrolanosterol, position 78 is always occupied by leucine. Therefore, the F78L substitution in the CYP51_Mt_ binding site was examined and found to slightly increase binding affinities toward compounds **8** and **10**, as opposed to the rest of the compounds whose binding affinities decreased (Fig. 2). Although a single amino acid substitution does not by any means convert bacterial protein into its mammalian counterpart, this finding is consistent with lack of toxicity in mammalian cells at inhibitory concentrations, and supports the possibility of rational design of highly selective anti-protozoan CYP51 inhibitors. The latter is of particular pharmacological importance as far as host-pathogen cross-reactivity is concerned, since CYP51 is present in human host.

The increased binding affinities toward CYP51_Tc_ of all the compounds we tested may indicate more extensive involvement of the BC-loop and C helix in protein-inhibitor interactions in CYP51_Tc_ than in CYP51_Mt_. Assuming that compound **8** binds CYP51_Tc_ in a similarly compact donut-like shape that fills the space adjacent to the porphyrin ring, its 300-fold increase in binding affinity could be achieved solely by stabilization of the BC-region of CYP51_Tc_ without engaging the species-specific cavity. This possibility opens the door to a rational design effort in which the beneficial features of both compounds **8** and **10** would be combined to yield third generation compounds that would more potently and selectively inhibit CYP51_Tc_. Toward this end compound **10** is currently being evaluated in animal models of Chagas' disease.
